# A comprehensive analysis of potential gastric cancer prognostic biomarker ITGBL1 associated with immune infiltration and epithelial–mesenchymal transition

**DOI:** 10.1186/s12938-022-00998-5

**Published:** 2022-05-20

**Authors:** Zhe Wang, Liu Fu, Junjie Zhang, Yanli Ge, Cheng Guo, Rui Wang, Min Deng, Qizhi Wang, Zhirong Wang

**Affiliations:** 1 Department of Gastroenterology, Tongji Hospital, Tongji University School of Medicine, 389 Xincun Road, Putuo District, Shanghai, 200065 People’s Republic of China; 2 Department of Gastroenterology, Putuo People’s Hospital, Tongji University, Shanghai, 200060 People’s Republic of China; 3 Department of Gastroenterology, The First Affiliated Hospital of Bengbu Medical College, Bengbu, Anhui 233004 People’s Republic of China

**Keywords:** Bioinformatics, Biomarker, Epithelial–mesenchymal transition, Gastric cancer, Immune infiltration, ITGBL1

## Abstract

**Background:**

Integrin, beta-like 1 (ITGBL1) is involved in a variety of human malignancies. However, the information on the involvement of ITGBL1 in gastric carcinoma (GC) is limited. Hence, this study aimed further to explore the functions and mechanisms of ITGBL1 in GC.

**Methods:**

First, multiple bioinformatics databases, including Oncomine, Tumor Immune Estimation Resource, UALCAN, and Kaplan–Meier Plotter, were used to predict the expression level and prognostic value of ITGBL1, as well as its association with immune infiltration and epithelial–mesenchymal transition (EMT) in GC. Quantitative reverse transcription–polymerase chain reaction and immunohistochemical analysis were used to detect the expression of ITGBL1 in both GC tissues and cells. Then, targeted silencing of ITGBL1 in GC cells was further used to examine the biological functions of ITGBL1.

**Results:**

These databases revealed that ITGBL1 was overexpressed and affected the overall survival in GC. Besides, the expression of ITGBL1 positively correlated with immune-infiltrating cells and EMT-related markers. Subsequently, molecular biology experiments verified these predictions. In GC tissues and cells, ITGBL1 was notably overexpressed. Loss-of-function studies showed that the knockdown of ITGBL1 significantly suppressed migration and invasion but promoted apoptosis in MGC803 GC cells. Furthermore, the inhibition of ITGBL1 resulted in remarkably increased protein expression levels of cadherin 1, while the expression of Vimentin, Snail, and transforming growth factor-β1 was downregulated, indicating the initiation and progression of GC caused by ITGBL1 partly via inducing EMT.

**Conclusions:**

To sum up, the findings indicated that ITGBL1 acted as a valuable oncogenic factor in GC.

## Introduction

According to Global Cancer Statistics 2020, gastric carcinoma (GC) is the fifth most common malignant tumor and fourth in terms of cancer-related mortality in the world [1]. Although substantial progress has been made on the pathogenesis and molecular biology of GC, approximately 80% of patients with GC do not receive the best treatments owing to locally advanced disease and metastasis at diagnosis [2]. In addition, the traditional therapeutic methods, such as surgery and chemotherapy, have limited value in advanced disease [3]. The occurrence and development of tumors are not only related to the pathological type and clinical stage but also closely interrelated to the abnormal expression of genes, which may serve as a biomarker for diagnosis and treatment [4]. Targeted therapy provides a new direction for the clinical treatment of tumors. Therefore, further exploration of GC is needed, including more potential biomarkers for early diagnosis and therapy.

Integrins are an important type of cell surface receptor via mediating the adhesion between cells and extracellular matrixes (ECM) to receive and conduct cascade signals and hence regulate cell survival, proliferation, movement, and other biological behaviors [5]. Increasing evidence indicates that numerous integrins are involved in the initiation and progression of tumors. For example, in esophageal squamous cell carcinoma, integrin β6 was observed in 41.6% of tumors compared with normal tissues, associated with histological differentiation, lymph node metastasis and Tumor Node Metastasis (TNM) stage [6]. Integrin ITGB3 interacted with heparan sulfate proteoglycans (HSPGs) and assisted in integrin endocytosis, promoting the metastasis of breast cancer [7]. In GC, integrin subunit alpha 11 promoted gastric cancer cell proliferation and invasion and inhibited apoptosis through the phosphatidylinositol 3-kinase/Akt pathway [8]. Integrin, beta-like 1 (ITGBL1), also called ten β Integrin EGF-like repeat domains (TIED), encodes beta integrin-related extracellular matrix protein, which is highly similar to integrin β subunits. ITGBL1 was first discovered from overlapping cDNA clones in 1999 [9]. Previous studies showed that ITGBL1 was dysregulated in several tumors and acted as either carcinogenic or tumor suppressor. For instance, the ITGBL1 level was elevated in colorectal carcinoma (CRC) and accelerated cell proliferation and migration [10]. Similarly, in hepatocellular cancer, high ITGBL1 expression was associated with a poor prognosis [11]. In contrast, ITGBL1 expression was downregulated in non-small cell lung cancer (NSCLC) and inhibited migration and invasion via binding to miR-576-5p [12]. Li et al. reported that ITGBL1 expression was upregulated and had a high positive correlation with distant metastasis and TNM stage in GC [13]. These studies suggested that ITGBL1 might be closely related to the development of tumors and played a guiding role in the diagnosis and prognosis of tumors. However, the functions and molecular mechanisms of ITGBL1 are still incompletely investigated in GC. Whether it can be used as a new target still needs to be explored.

In the present study, we first used bioinformatics repositories including Tumor Immune Estimation Resource (TIMER) (http://timer.cistrome.org/) [14], Oncomine (https://www.oncomine.org/) [15] and UALCAN (http://ualcan.path.uab.edu) [16] databases to predict the expression level of ITGBL1 and found that it was upregulated in GC tissues compared with normal tissues. Subsequently, the finding was verified by quantitative reverse transcription–polymerase chain reaction (qRT–PCR) and immunohistochemical (IHC) analysis with fresh clinical samples collected. Next, we performed a comprehensive analysis including the overall survival (OS) of ITGBL1 and the association between ITGBL1 expression and immune infiltration and epithelial–mesenchymal transition (EMT) using diverse public databases Kaplan–Meier Plotter (http://www.kmplot.com/) [17], Tumor, Immune System Interaction DataBase (TISIDB) (http://cis.hku.hk/TISIDB/) [18] and Starbase online server (http://www.sysu.edu.cn/) [19]. Finally, functional and mechanical assays were conducted to further investigate the biological behavior of ITGBL1 and confirm the correlation between ITGBL1 and EMT. It was concluded that ITGBL1 might play a major role in the carcinogenicity of GC and serve as a potential marker in targeted therapy.

## Results

### ITGBL1 high expression detected GC tissues by bioinformatics analysis

**Fig. 1:**
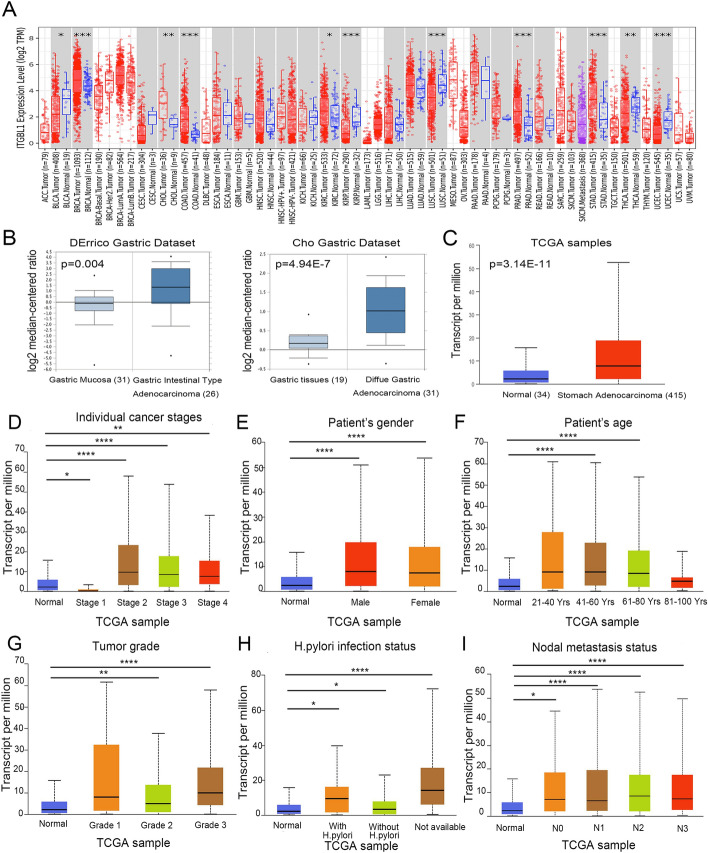
ITGBL1 levels and clinical factors associated with ITGBL1 expression in GC tissues via bioinformatics analysis. **A** mRNA expression of ITGBL1 of different tumors in the Timer database. **B** mRNA levels of ITGBL1 significantly increased in GC tissues compared with normal tissues based on the Oncomine database. **C** In UALCAN, ITGBL1 mRNA expression was upregulated in stomach adenocarcinoma tissues compared with normal tissues. **D**–**I** ITGBL1 expression in subgroups of patients with GC based on cancer stage, sex, age, tumor grade, *H. pylori* infection status, and nodal metastasis status. ^*^P < 0.05, ^**^P < 0.01, ^****^P < 0.0001

First, we analyzed ITGBL1 expression in various tumors using the TIMER database, which revealed the dysregulation of ITGBL1 in different tumors (Fig. 1A). Subsequently, a public database Oncomine was used to investigate the levels of ITGBL1 mRNA in GC tissues. The world’s largest oncogene chip database was used to analyze the data uploaded from DErrico and Cho gastric data set samples. DErrico data set has 31 gastric mucosa samples and 26 gastric intestinal-type adenocarcinoma samples, and Cho gastric data set contains 19 gastric tissues and 31 diffuse gastric adenocarcinoma tissues. These unpaired data showed that mRNAs of ITGBL1 were overexpressed in both gastric intestinal-type adenocarcinoma (*P* = 0.004, fold change: 2.659) and diffuse gastric adenocarcinoma (*P* = 4.94e−7, fold change: 1.781) than those in normal tissues by comparing the mean value of log2 median-centered ratio (Fig. 1B). Consistent with this, The Cancer Genome Atlas (TCGA) gene expression data available in an online web tool UALCAN were used for analyzing cancer omics data. The change in unpaired data in the ITGBL1 transcription level in stomach adenocarcinoma tissues was 3.5-fold relative to that in unpaired normal tissues (*P* = 3.14e−11, Fig. 1C). In addition, in the public UALCAN website, various clinicopathological characteristics, including cancer stages, patient’s sex, patient’s age, tumor grade, *Helicobacter pylori* infection status, and nodal metastasis status of GC samples in the TCGA were further analyzed. The results consistently showed that the mRNA expression level of ITGBL1 was significantly higher in patients than in healthy individuals based on subgroups, including cancer stages 2–4, sex, age 41–80 years, tumor grades 2–3, *H. pylori* infection status, and nodal metastasis status with statistical significance (Fig. 1D–I). Thus, it was concluded that ITGBL1 was abnormally expressed in various tumors including GC.

### Upregulated ITGBL1 in GC tissues with experimental verification

**Fig. 2:**
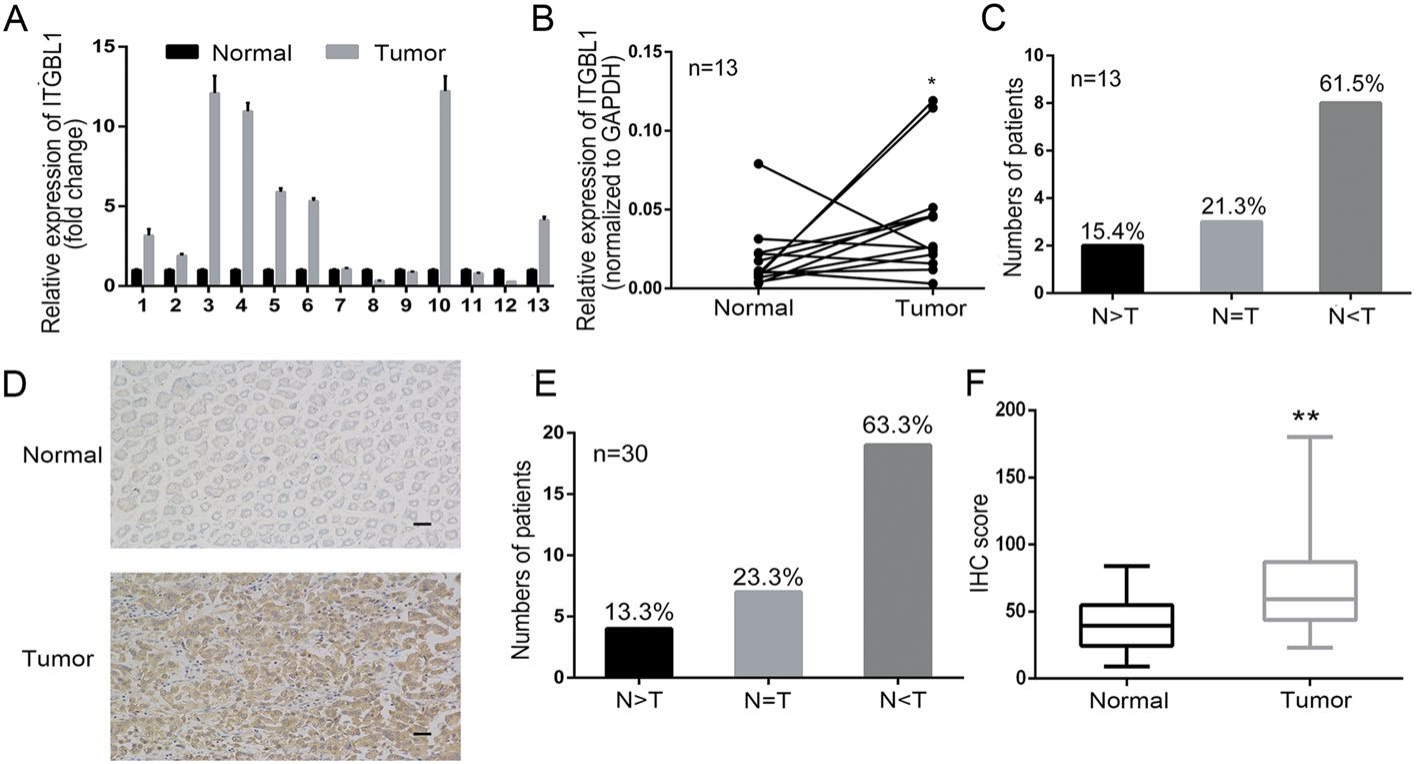
Experimental verification of ITGBL1 expression level. **A**–**C** qRT–PCR was conducted to detect ITGBL1 mRNA expression in 13 GC samples as fold change (A) and normalized to GAPDH expression (**B**). Results showed that 61.5% (8/13) of patients with GC had high expression of ITGBL1 (**C**). **D**, **F** Immunohistochemical staining of ITGBL1 for 30 paired patients with GC. Representative images of normal and tumor tissues are shown; scale bar: 10 µm (**D**); 63.3% of patients with ITGBL1-positive expression in GC tissues (**E**). Results of ITGBL1 IHC scores in GC compared with paired nontumor tissues (**F**). ^*^P < 0.05, ^**^P < 0.01

Based on bioinformatics findings, we conducted experimental confirmation subsequently. A total of 13 paired GC tissues were collected for qRT–PCR to verify the expression level of ITGBL1. The results indicated that ITGBL1 was often overexpressed in GC tissues compared with matched adjacent normal tissues. In eight pairs of matched samples, the expression of ITGBL1 increased in tumor tissues relative to adjacent nontumor tissues, accounting for 61.5% (8/13 patients); however, only two patients had low expression in GC tissues (Fig. 2A–C, *P* < 0.05). In addition, the translational level of ITGBL1 was measured by IHC staining in 30 paired GC tissues. Representative images are displayed in Fig. 2D. The results revealed that the ITGBL1 protein level remarkably increased in GC tumors with 19 out of 33 patients exhibiting higher ITGBL1 protein levels relative to the adjacent nontumor tissues (Fig. 2D, E). IHC analysis performed with these paired tissues showed more ITGBL1-positive tissues in tumor tissues than in nontumor tissues (Fig. 2F, *P* < 0.01). In summary, these results indicated that both the mRNA and protein levels of ITGBL1 were highly expressed in GC.

### Higher expression of ITGBL1 with poorer prognosis in GC

**Fig. 3:**
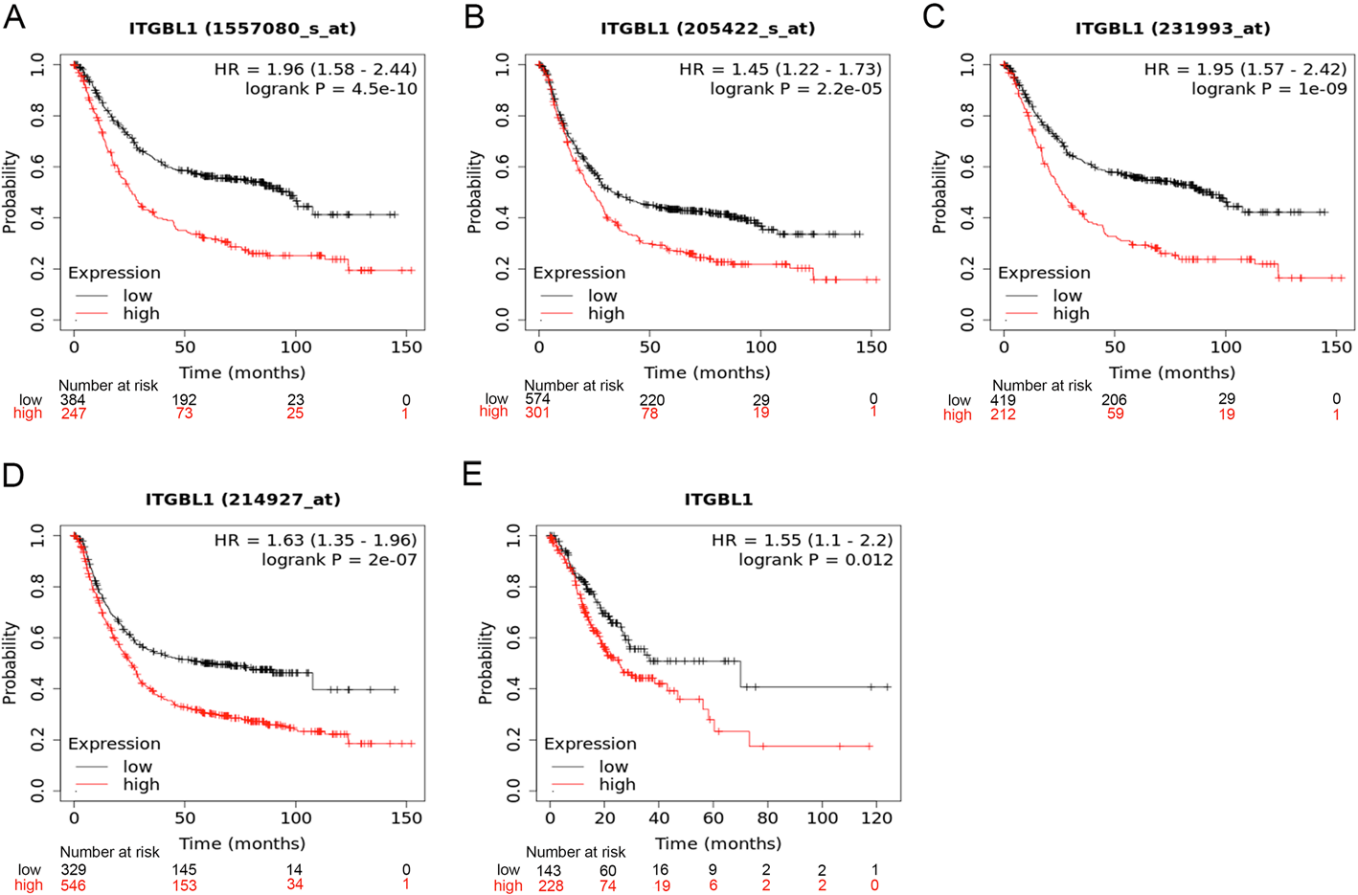
Prognostic value of ITGBL1. **A**–**D** Overall survival of ITGBL1 in different gene chips based on the Kaplan–Meier Plotter. **E** In the Kaplan–Meier Plotter, the prognosis of ITGBL1 is shown with RNA sequencing

For predicting the clinical prognosis of ITGBL1, the Kaplan–Meier Plotter was used to analyze the OS in different microarray chips and RNA-seq data. Four microarray chips commonly indicated that high ITGBL1 was closely related to poor OS (Fig. 3A–D). Consistent with this, RNA-seq results showed that upregulated ITGBL1 was significantly associated with the poor OS of patients with GC (Fig. 3E). Overall, these findings revealed that ITGBL1 might serve as a potential diagnostic and prognostic indicator of GC.

### ITGBL1 level was associated with the number of immune-infiltrating cells in GC

**Fig. 4:**
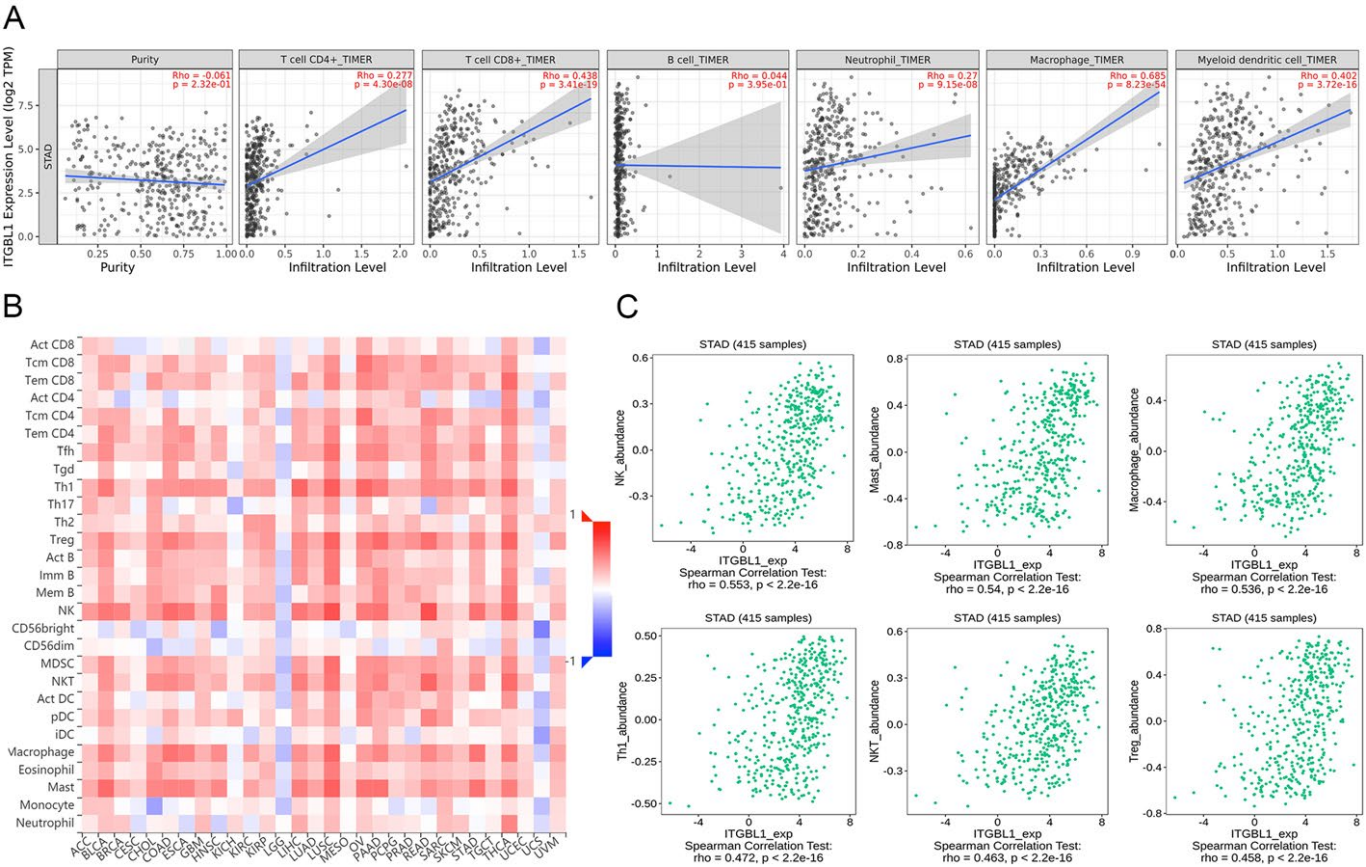
Association of ITGBL1 with immune-infiltrating cells. **A** TIMER database was used to predict the correlation between ITGBL1 and different immune cells (CD4 + T cells, CD8 + cells, neutrophils, macrophages, B cells, and myeloid dendritic cells). **B** Hot plot showed the relations between the abundance of immune-related signatures of 28 TILs and ITGBL1 expression in diverse tumors. **C** Top six immune cells with a strong correlation with ITGBL1

**Fig. 5:**
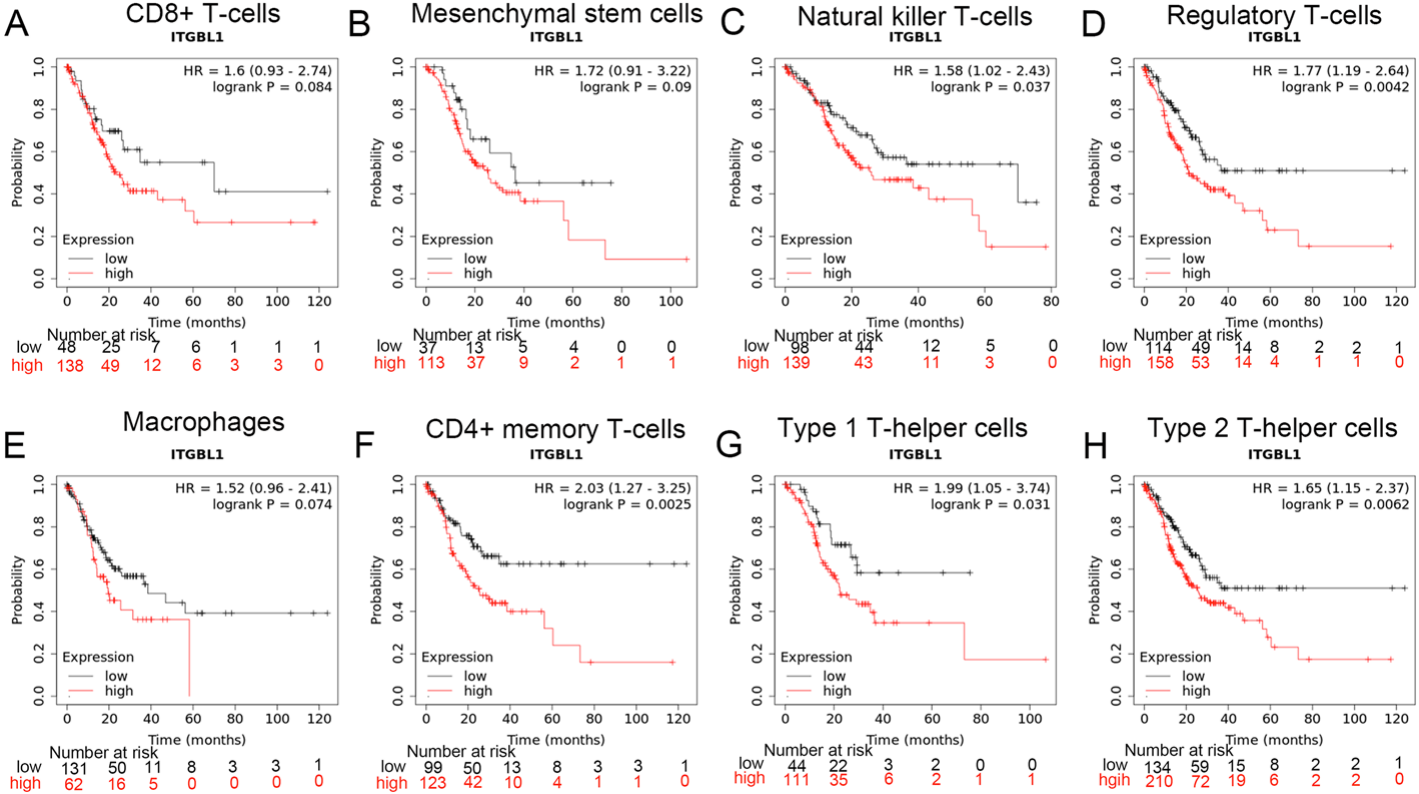
Prognosis of ITGBL1 in enriched natural killer T cells, regulatory T cells, CD4 + memory T cells, Type 1 T helper cells, Type 2 T helper cells, CD8 + T cells, mesenchymal stem cells, and macrophages

The TIMER database was used to predict the relationship of the ITGBL1 level with the number of immune-infiltrating cells in GC. The results showed that ITGBL1 was significantly positively associated with the number of CD4 + T cells (*r* = 0.277, *P* = 4.30e−08), CD8 + T cells (*r* = 0.438, *P* = 3.41e−19), neutrophils (*r* = 0.27, *P* = 9.15e−08), macrophages (*r* = 0.685, *P* = 8.23e−54), and myeloid dendritic cells (*r* = 0.402, *P* = 3.72e−16). However, no obvious correlation was found between the ITGBL1 level and the number of B cells (*r* = 0.044, *P* = 3.95e−01) (Fig. 4A). In addition, we performed the TISIDB database analysis to explore the association between the ITGBL1 level and number of tumor-infiltrating lymphocytes (TILs). In GC, the number of 19 immune-infiltrating cells positively correlated with the expression of ITGBL1 (Fig. 4B). Notably, the top six cells were natural killer cells (*r* = 0.553, *P* < 2.2e−16), mast cells (*r* = 0.54, *P* < 2.2e−16), macrophages (*r* = 0.536, *P* < 2.2e−16), Type 1 T helper cells (*r* = 0.472, *P* < 2.2e−16), natural killer T cells (*r* = 0.463, *P* < 2.2e−16), and regulatory T cells (*r* = 0.458, *P* < 2.2e−16), which displayed a moderate relationship (Fig. 4C). Subsequently, we explored the impact of immune cells on the prognosis of ITGBL1 using the Kaplan–Meier Plotter. We found that for natural killer T cells (*P* = 0.037), regulatory T cells (*P* = 0.0042), CD4 + memory T cells (*P* = 0.0025), Type 1 T helper cells (*P* = 0.031), and Type 2 T helper cells (*P* = 0.0062), higher ITGBL1 expression predicted poor OS. However, no significant correlations were found with abundant CD8 + T cells (*P* = 0.084), mesenchymal stem cells (*P* = 0.09), and macrophages (*P* = 0.074) (Fig. 5). Thus, our findings suggested that ITGBL1 might be involved in regulating immune infiltration and influenced the prognosis of GC partly through immune infiltration.

### Silencing of ITGBL1 expression promoted GC cell MGC803 apoptosis and suppressed MGC803 cell migration and invasion in vitro

**Fig. 6:**
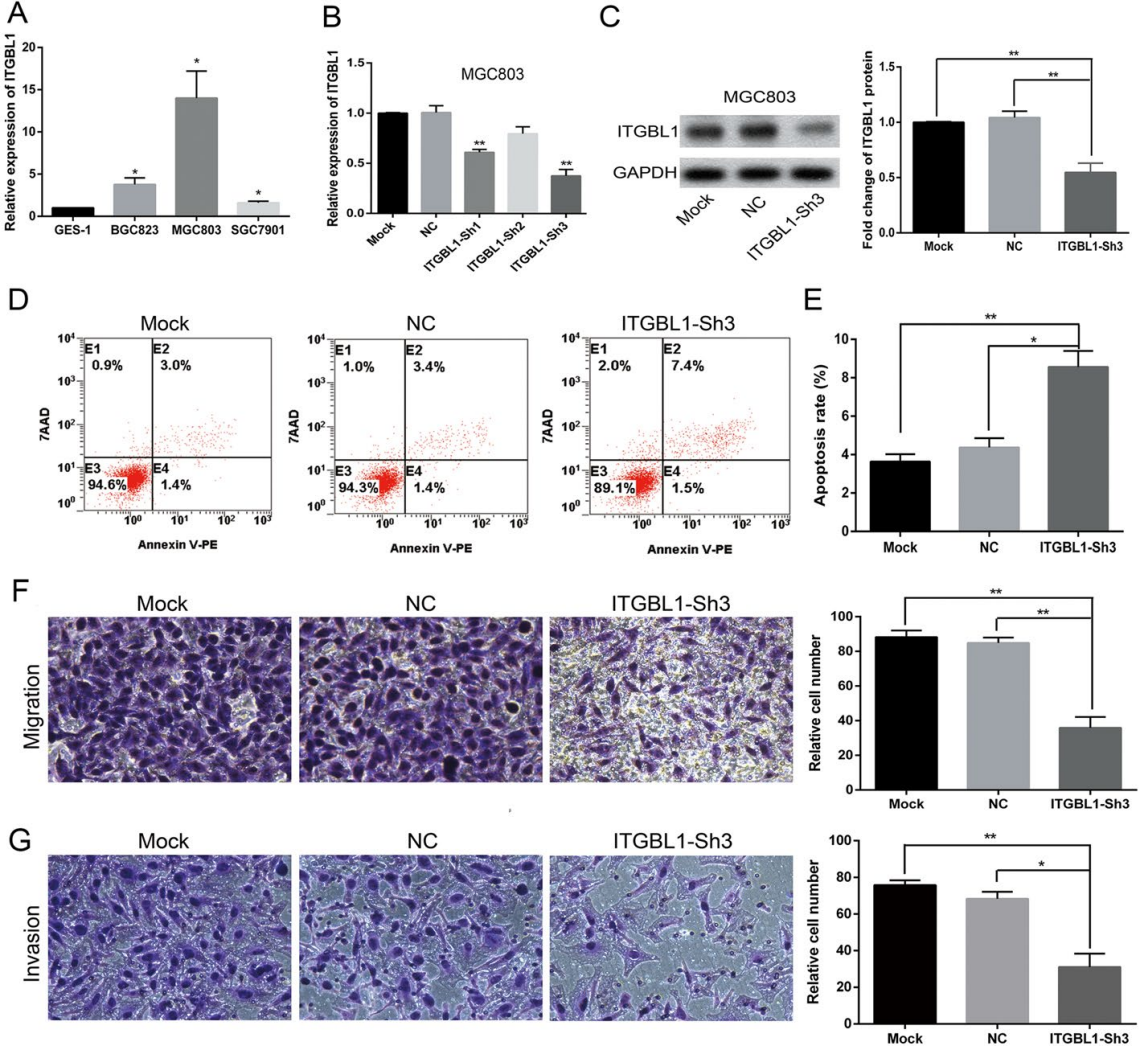
Expression of ITGBL1 in GC cells and silencing of ITGBL1 promoted apoptosis and inhibited the migration and invasion of MGC803 cells*.* **A** Results of qRT–PCR for ITGBL1 mRNA expression in GC cell lines relative to GES-1. **B**, **C** Results of qRT–PCR and Western blot for the silencing of ITGBL1. GAPDH was used as an internal control. **D**, **E** Representative images and quantification results for flow cytometry to evaluate apoptosis induction by ITGBL1 in MGC803 cells transfected with or without ITGBL1–Sh3. **F** Representative images for Transwell assay and quantification results of Transwell migration. Migrated cells were analyzed using ImageJ. **G** Representative images of Transwell invasion assay and quantitative analysis of invasive cells using Image J. The results showed that the knockdown of ITGBL1 suppressed cell migration and invasion. ^*^P < 0.05, ^**^P < 0.01

The ITGBL1 expression levels in an immortalized normal gastric epithelial cell line GES-1 and three GC cell lines (BGC823, SGC7901, and MGC803) were compared. The results showed that ITGBL1 had relatively increased expression in all GC cell lines, especially in MGC803, with a fold change of up to 12 relative to that in GES-1 (Fig. 6A). Therefore, MGC803 was chosen for investigating the knockdown of ITGBL1. Next, three specific shRNAs targeting ITGBL1 were designed and transfected into MGC803 cells. The qRT–PCR analysis revealed that ITGBL1–Sh3 mRNA levels were successfully and effectively knocked down compared with mock (without shRNA transfection) and NC (with negative control shRNA transfection), from a baseline of 1.00 to 0.38 in MGC803 cells (*P* = 0.0026, Fig. 6B). Subsequently, the ITGBL1 protein level was effectively downregulated compared with that in mock and NC cells (*P* = 0.0059 and 0.0083, respectively) in MGC803 cells (Fig. 6C). Thus, ITGBL1–Sh3 cells were remarkably constructed and used in all subsequent experiments. Flow cytometry assay was conducted to examine whether the knockdown of ITGBL1 expression caused programmed cell death. Relative to transfection with or without NC (4.37 ± 0.48 and 3.63 ± 0.38) %, the inhibition of ITGBL1 expression remarkably increased cell apoptosis in MGC803 cells (8.57 ± 0.83) % (*P* = 0.0118 and 0.0056, respectively; Fig. 6D, E). Next, the involvement of ITGBL1 in the migration and invasion of GC cells was further characterized using Transwell assay, revealing that ITGBL1 silencing resulted in a significant decrease in the number of migrating MGC803 cells versus mock and NC cells (*P* = 0.002 and 0.0021, respectively; Fig. 6F). Similarly, the invasion ability was inhibited by transfection with ITGBL1–Sh3 compared with transfection with NC or mock (*P* = 0.0046 and 0.0107, respectively; Fig. 6G).

### ITGBL1 involvement in EMT in GC cells

**Fig. 7:**
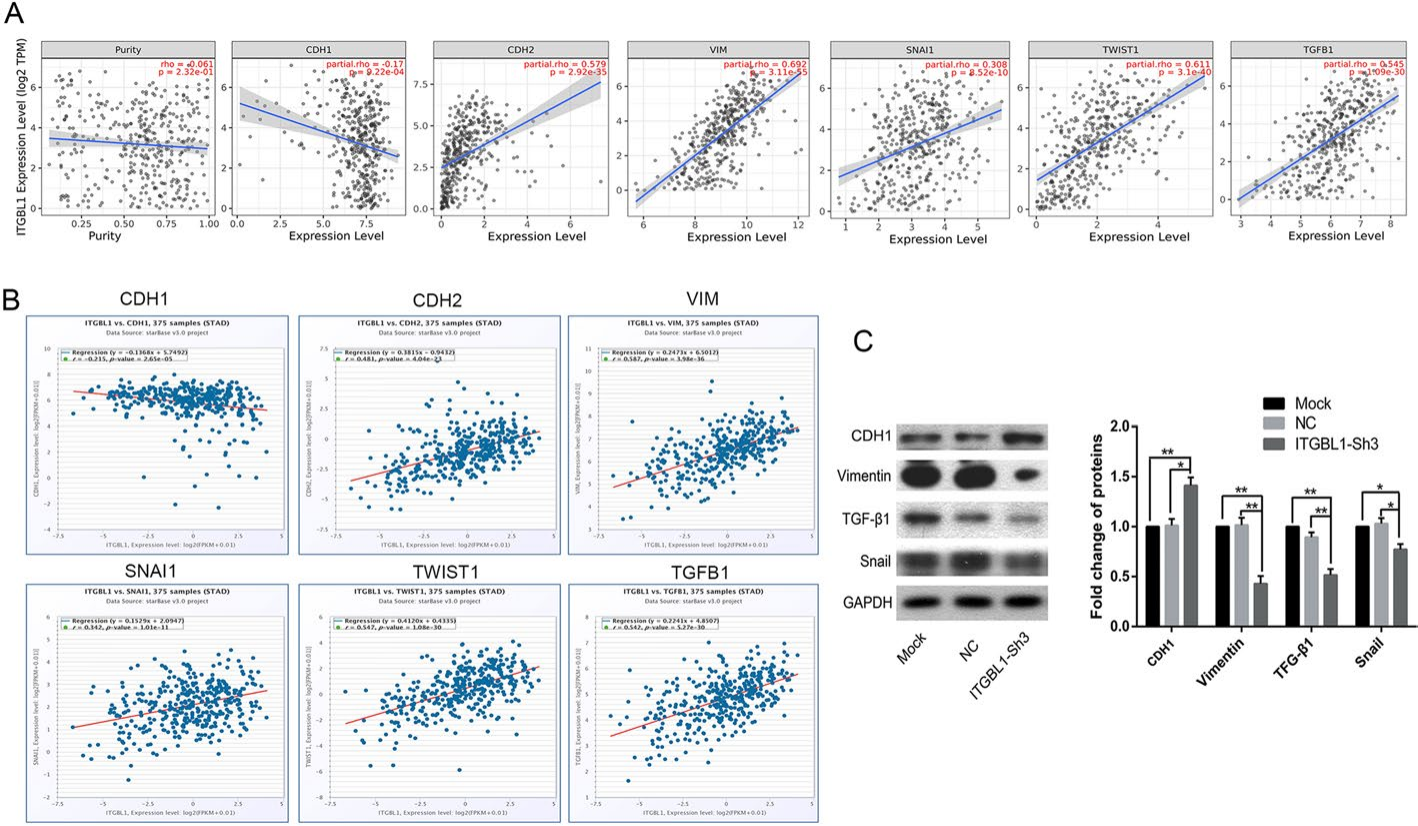
ITGBL1 was involved in the EMT process in MGC803 cells. **A** Online TIMER database was used to explore the correlation between the expression of ITGBL1 and EMT-related factors. **B** Starbase further predicted the relationship of between ITGBL1 and CDH1, CDH2, Vim, Snai1, Twist1, and TGF-β1. **C** Representative images and quantitative Western blot analysis of the expression of CDH1, Vimentin, Snail, and TGF-β1 proteins in MGC803 cells treated with mock or NC or silencing of ITGBL1. ^*^P < 0.05, ^**^P < 0.01

The TIMER database was used to explore the correlations between ITGBL1 and EMT-related factors including CDH1, CDH2, Vimentin (VIM), SNAI1, TWIST1, and TGF-β1. The predicted results showed that ITGBL1 expression was negatively associated with CDH1 mRNA expression (*R* =  − 0.17, *P* = 9.22e−04) but had a positive association with the transcript levels of CDH2 (*R* = 0.579, *P* = 2.92e−35), Vimentin (*R* = 0.692, *P* = 3.11e−55), Snai1 (*R* = 0.308, *P* = 8.52e−10), TWIST1 (*R* = 0.611, *P* = 3.1e−40), and TGF-β1 (*R* = 0.545, *P* = 1.09e−30) (Fig. 7A). Besides, another database, Starbase online server, was used, and the trend of relevance was consistent. The ITGBL1 level strongly positively correlated with mRNAs of CDH2 (*R* = 0.481, *P* = 4.04e−23), VIM (*R* = 0.587, *P* = 3.98e−36), SNAI1 (*R* = 0.342, *P* = 1.01e−11), TWIST1 (*R* = 0.547, *P* = 1.08e-30), and TGF-β1 (*R* = 0.542, *P* = 5.27e-30), but significantly negatively correlated with CDH1 (*R* =  − 0.215, *P* = 2.65e−05) (Fig. 7B). After predicting, the expression of EMT biomarkers and transcription factors was investigated using the Western blot analysis to further identify the relationship between ITGBL1 and EMT in MGC803 cells. The results showed that the inhibition of ITGBL1 led to a distinct enhancement in the protein expression of epithelial biomarker CDH1. In contrast, the expression of mesenchymal biomarker Vimentin and main transcription factors Snail and TGF-β1 was remarkably downregulated in ITGBL1–Sh3 cells compared with mock and NC cells (Fig. 7C). The results elucidated that the inhibition of ITGBL1 in part suppressed the process of EMT.

## Discussion

Integrin is one of the important members of the cell adhesion molecule family, comprising a heterodimer formed by α and β subunits. It is a group of transmembrane glycoprotein receptors widely distributed on the cell surface, not only adjusting cellular morphology, polarization, and motility but also regulating disease progression through adhesion to cells and ECM and signal transduction functions [20]. ECM provides a suitable microenvironment for tumor survival and activity and is involved in tumor barrier function. Previous studies showed that ECM molecules such as periostin and dermatopontin were important in tumor initiation and progression [21, 22]. Recently, ITGBL1, as an ECM-related protein, has been found to be dysregulated in malignant tumors; however, the function and biological mechanism of ITGBL1 still remains unclear. In the present study, ITGBL1 expression was upregulated in both GC tissues and GC cells. Hence, ITGBL1 might serve as an innovative prognostic biomarker and therapeutic target for patients with GC.

The ITGBL1 gene is located at 33.1 on the long arm of human chromosome 13, with a total length of 26 kb; it can encode an osteoblast-specific cysteine-rich protein with a length of 494 amino acids. Accumulating evidence indicated that ITGBL1 was crucial in carcinogenesis and cancer progression. Multiple studies showed that ITGBL1 functioned as an oncogene [23, 24]. For example, in acute myeloid leukemia (AML), ITGBL1 methylation was reported to impact prognosis, and patients with hypermethylation of ITGBL1 tended to have leukemia-free survival and shorter OS [25]. Similarly, high expression of ITGBL1 facilitated cell migration and adhesion in ovarian cancer through Wnt/planar cell polarity (PCP) and focal adhesion kinase (FAK) /SRC signaling pathways [26]. Moreover, higher expression of ITGBL1 was more likely to cause tumorigenesis via Akt signaling in GC [27]. In contrast, ITGBL1 functioned as a tumor suppressor in NSCLC, inhibiting cell migration and invasion [12]. A previous study reported based on integrated bioinformatics analysis that ITGBL1 was a prognostic factor [28]. However, the biological mechanism of the dysfunction of ITGBL1 has not been fully understood. The present study showed using public databases TIMER, Oncomine and UALCAN that ITGBL1 was highly expressed in GC tissues compared with adjacent normal tissues. Subsequently, the high expression of ITGBL1 in GC tissues compared with normal tissues was confirmed in 43 pairs of GC tissues using IHC staining. Besides, ITGBL1 in GC tissues derived from TCGA samples was significantly highly expressed in subgroups including cancer stage, tumor grade, and lymph node metastasis compared with normal gastric tissues. In addition, higher ITGBL1 expression was closely associated with poorer OS in patients with GC using Kaplan–Meier statistical analysis, which indicated that ITGBL1 might serve as an independent prognostic factor in GC. The expression of ITGBL1 in GC cells and normal gastric mucosa epithelial cells was detected at the mRNA and protein levels using qRT–PCR and Western blot, respectively. The results showed that the mRNA and encoded protein expression of the ITGBL1 gene significantly increased in GC cells, especially in MGC803 cells, which was consistent with its expression in tissues. Furthermore, the cell apoptosis assay and Transwell migration/invasion assay results indicated that silencing ITGBL1 suppressed MGC803 cell migration and invasion but promoted apoptosis. In summary, the high expression of ITGBL1 significantly correlated with the survival and prognosis of patients with GC, and played an important role in the migration and invasion of GC cells.

Immune infiltration plays a decisive role in tumorigenesis and is one of the critical mechanisms for tumor initiation and progression. Immune-infiltrating CD4 + T cells are involved in anti-tumor function, which not only contributes to the activation of CD8 + T cells but also helps in the generation and preservation of memory cytotoxic T lymphocyte responses [29]. Neutrophils promote gastric cancer progression by reducing T-cell immunity [30]. Cheli et al. [31] reported that ITGBL1, as a novel immunomodulator, promoted melanoma development by inhibiting the cytotoxicity of natural killer cells both in vitro and in vivo. However, the relationship between ITGBL1 and immune infiltration in GC remains unclear. Therefore, this study further used the TIMER and TISIDB databases to analyze the correlation between ITGBL1 expression and immune infiltration in GC tissues. We found that ITGBL1 had a close relationship with CD4 + T cells, CD8 + T cells, neutrophils, macrophages, myeloid dendritic cells, natural killer cells, mast cells, Type 1 T helper cells, natural killer T cells, and regulatory T cells. In addition, the Kaplan–Meier Plotter website predicted that higher ITGBL1 expression predicted poor OS in enriched natural killer T cells, regulatory T cells, CD4 + memory T cells, Type 1 T helper cells, and Type 2 T helper cells. These findings revealed that ITGBL1 might regulate the progression of GC by affecting the interaction between immune cells and malignant tumor cells. ITGBL1 reflected the immune status of GC in part and provided notable insights into GC immunotherapy. Based on this, further experimental studies will be carried out to clarify the specific mechanism of action.

EMT phenotype is a transient and reversible procedure by which a polarized, epithelial phenotype switches to an elongated mesenchymal phenotype, including the downregulation of E-cadherin expression and the upregulation of N-cadherin expression, resulting in increased migration and invasion [32]. A substantial body of evidence implied that EMT was significantly associated with metastasis and prognosis of various cancers, including GC [33]. Previous studies confirmed that the regulation of EMT was a complex network including multiple signaling pathways, such as Wnt/β-catenin, Notch, hepatocyte growth factor, and TGF-β [34–36]. Among these, the TGF-β signaling pathway was the major contributor, regulating a series of cellular processes including intercellular substance production, differentiation, apoptosis, immune reaction, and inflammatory response [37]. Studies found that during EMT in cells, TGF-β can enhanced the expression of integrins and made them more exposed on the cell membrane surface, thereby regulating cell–matrix interactions. As a cytokine receptor, integrins and their kinases were also necessary for the activation of TGF-β and its induction of cellular EMT [38]. The occurrence of EMT was closely related to multiple members of the integrin family. For example, the TGF-β signaling pathway functioned as a key regulator of fibrogenesis, and elevated expression of ITGBL1 promoted HBV-related liver fibrogenesis by interacting with TGF-β1 [39]. In CRC, ITGBL1 levels strongly correlated with EMT-associated genes, serving as a crucial indicator of an EMT phenotype [40]. High expression of ITGBL1 facilitated bone metastasis via inducing the TGF-β signaling pathway in breast cancer [41]. Li et al. found that high expression of ITGBL1 promoted invasion and migration and activated EMT in prostate cancer [42]. The present study found that the expression levels of EMT-related biomarker CDH1 increased, while the expression of biomarker Vimentin and EMT transcription factor Snail decreased after the downregulation of ITGBL1. In addition, the protein expression level of EMT-stimulating factor TGF-β decreased after the silencing of ITGBL1, suggesting that ITGBL1 could promote EMT in part by activating the TGF-β signaling pathway in GC. In addition, the online TIMER and Starbase analyses yielded consistent results that ITGBL1 expression correlated negatively with CDH1 expression, but correlated positively with VIM, SNAI1, TWIST1 and TGF-β1 expression. These results further demonstrated that ITGBL1 regulated the biological functions of GC partly via inducing EMT signaling pathways.

However, this study had certain limitations. First, the patient samples were insufficient for IHC analysis of GC tissues. Besides, we analyzed the relationship between ITGBL1 and immune infiltration through bioinformatics, which still required follow-up experimental validation. Although we found that ITGBL1 induced EMT via the TGF-β signaling pathway, whether the ITGBL1 directly acted on the TGF-β receptor or regulated the TGF-β signaling pathway through other molecules was not determined. Therefore, a follow-up study will be devoted to expanding the number of GC samples and exploring the specific mechanism of the TGF-β signaling pathway regulated by ITGBL1, and the effect of ITGBL1 on immune infiltration in promoting the development of GC.

## Conclusions

Based on public bioinformatics analysis and biological experiments, ITGBL1 was predicted and found to be highly expressed in GC and associated with immune infiltration and EMT. Moreover, one of the biological functions of ITGBL1 was to enhance the migration and invasion capabilities of MGC803 cells. In summary, these findings provided an insight that ITGBL1 served as an important diagnostic and prognostic tumor biomarker and a potential therapeutic target for GC, and hence was worth exploration.

## Materials and methods

### ITGBL1 expression in multiple databases

To evaluate the expression level of ITGBL1 in somatic tumors, Oncomine TIMER, and UALCAN public databases were used first. The Oncomine database is an online website containing Gene Expression Omnibus (GEO) and TCGA integration for endogenous mRNA levels. The TIMER server is an integrated resource mainly used to analyze immune-infiltrating cells, but it also allows researchers to estimate the differential expression for genes of interest between tumors and adjacent normal tissues across diverse cancer types. The UALCAN database was used to compare the expression level of ITGBL1 not only in stomach adenocarcinoma and normal tissues but also in different clinical classification subgroups including cancer stages, sex, age, tumor grade, HP infection status, and nodal metastasis status. The distributions of gene expression levels are displayed using box plots.

### Survival analysis

Kaplan–Meier Plotter is a website for online survival analysis involving a variety of tumors for GEO, European genome–phenome archive, and TCGA databases, which was used to evaluate the OS rate of gastric cancer tissues based on high-throughput sequencing data or gene chips.

### Prediction of association between ITGBL1 and tumor-infiltrating immune cells

The relationship between six immune infiltrates (B cells, CD4 + T cells, CD8 + T cells, neutrophils, macrophages, and dendritic cells) and ITGBL1 was estimated using the TIMER public website. Besides, another immune-related web server, TISIDB, was used to analyze the association between the abundance of TILs and expression of ITGBL1 by comparing the Spearman correlation between ITGBL1 expression and TILs in human cancers.

### Prediction of correlation between ITGBL1 and EMT-related factors

TIMER and Starbase web servers were used to assess the correlation between EMT-related factors and ITGBL1 expression levels in gastric cancer tissues. The two databases contained the routine data analysis of the mRNA of any gene of interest in the TCGA, including differential expression, survival analysis, and co-expression. The degree of correlation was computed using purity-adjusted partial Spearman's rho value. The correlations of ITGBL1 with these factors were finally shown with scatter plots.

### Tissue specimens

The study included 43 matched tumor samples from patients with surgically resected GC from the First Affiliated Hospital of Bengbu Medical College in 2017 for clinical indications. This study was authorized by the research medical ethics committee of the First Affiliated Hospital, Bengbu Medical College and Tongji Hospital, Tongji University. Each patient signed an informed consent form. All tissue specimens were pathologically confirmed as GC.

### Reverse transcription and quantitative real-time polymerase chain reaction

Total RNA was extracted with TRIzol reagent (Life Technologies, CA, USA). Subsequently, cDNA was synthesized by reverse transcription with PrimeScript RT kits (Takara Biotechnology Co., Japan) following the manufacturer’s protocol. Subsequently, generated cDNA was amplified with an SYBR PrimeScript RT-PCR kit (Takara Biotechnology Co.) on an ABI 7900 Real-Time PCR system. The primers used in the study were as follows: ITGBL1, (sense) 5′-AGACCTACGACGGGAGCAC-3′ and (antisense) 5′-ACCTGCATTAGAGCAGATGATGT-3′; GAPDH, (sense) 5′-ATCACCATTGGCAATGAG-3′ and (antisense) 5′-AAGGTAGTTTCGTGGATG-3′. Three-step cycling was adopted, and the reaction conditions were as follows: 95 °C for 30 s, 60 °C for 30 s, and 72 °C for 1 min; the number of cycles was 30. After the reaction, the PCR products were placed in a –20 °C refrigerator for subsequent experiments. The 2^−comparative Ct^ (2^−ΔΔCt^) formula was used to calculate the relative expression levels of ITGBL1, and GAPDH was normalized as an internal control.

### IHC staining

Tissues from patients with GC were fixed with 10% formalin for 48 h and then embedded with paraffin. Followed by deparaffinization and rehydration, the tissues were treated with 3% H_2_O_2_ for 10 min prior to blocking in goat serum for 1 h. Subsequently, a specific primary antibody ITGBL1 (1:100, Proteintech, China) was used to incubate the sections at 4 °C overnight. Next, horseradish peroxidase-labeled secondary antibodies were used to incubate tissue sections for 1 h at 37 °C and analyzed by the streptavidin − biotin complex method. The staining intensity was arranged as follows: 0, negative; 1, weak; 2, strong. IHC scoring was based on the percentage of positive cells multiplied by staining intensity. The formula was as follows: IHC score = 1 × (percent weakly stained cells) + 2 × (percentage of strongly stained cells). The evaluation of color development results was performed as previously described [43].

### Cell culture

Human normal gastric epithelial cell line GES-1 and GC cell lines SGC7901, BGC823, and MGC803 were provided by the Cell Division Center in Tongji Hospital of Tongji University, Shanghai, China. They were cultured in 90% Dulbecco’s Modified Eagle medium (DMEM, Gibco) with 10% fetal bovine serum (FBS, Gibco) at 37 °C and incubated in a humidified 5% CO_2_ atmosphere.

### Oligonucleotide transfection

Furthermore, 2.0 × 10^5^ cells/well of MGC803 cell lines were seeded in a six-well plate prior to transfection. After reaching 50% fusion density, the cells were transfected with an ultimate concentration of 50 nM of either negative control (NC) or ITGBL1-sh (targeted to interfere with ITGBL1) using Lipofectamine 2000 Reagent (Thermo Fisher Scientific, MA, USA). Three different oligonucleotide chains specifically targeting ITGBL1 were designed by Asia-Vector Biotechnology (Shanghai, China). The sequences were as follows: (sense) ITGBL1-sh1:5′-TGGGAAGTGTTACTGTGGA-3′, (antisense) 5′-TCCACAGTAACACTTCCCA-3′, (sense) ITGBL1-sh2: 5′-ACGATGAAACAGAAGAAAT-3′, (antisense) 5′-ATTTCTTCTGTTTCATCGT-3′, ITGBL1-sh3: (sense) 5′-GTGGACTTGTGTATGGTAA-3′, (antisense) 5′-TTACCATACACAAGTCCAC-3′. After 6 h of transfection, the culture medium was aspirated, a fresh medium containing 10% serum was added, and the culture was continued for 24 or 48 h, following which the cells were collected for subsequent cell function experiments.

### Cell apoptosis assay

An Annexin V–PE/7-aminoactinomycin D (7-AAD) apoptosis detection kit (Yeasen, China) was used for measuring cell apoptosis. Transfected cells were reseeded at a density of 5 × 10^5^ cells/well onto six-well plates and collected with 0.25% trypsin without EDTA. Next, the collected cells were washed twice with cold phosphate buffer saline (PBS) prior to resuspending with 1× binding buffer. After that, 5 μL of Annexin V–PE and 10 μL of 7-AAD (Yeasen, China) were added to each cell suspension to stain cells and incubated for 30 min. After adding 385 µL of 1× Annexin-binding buffer, the cell apoptosis was assessed by flow cytometry.

### Transwell migration/invasion assay

For assessing migration, the cells were cultured in the upper compartment of a Transwell chamber of an 8-μm pore size insert (Corning, NY, USA) at a density of 1.5 × 10^4^ cells/well in serum-free culture medium. For evaluating invasion, the cells were incubated in the upper chamber of the insert coated with Matrigel (Corning, NY, USA). The lower chambers of both experiments were treated with DMEM containing 20% FBS. Following 48 h incubation, the cells remaining on the upper compartment were wiped off with cotton swabs. The cells that migrated and invaded the bottom chamber were stained with crystal violet for 30 min. The results of the quantitative analysis were assessed using ImageJ software.

### Western blot analysis

Bicinchoninic acid (BCA) protein assay was performed to determine protein concentrations. Equal amounts of protein lysates were segregated with 10% polyacrylamide gels (Bio-Rad, CA. USA) and transferred to 0.45 µm nitrocellulose membranes (Bio-Rad). Then, 5% BSA was used to block membranes for 1 h prior to incubation with primary antibodies: ITGBL1 (1:1000, Proteintech, China), Vimentin (1:1000, Cell Signaling, MA, USA), E-cadherin (1:1000, Proteintech, China), Snail (1:1000, Cell Signaling), TGF-β1 (1:2000, Abcam, China), and glyceraldehyde-3-phosphate dehydrogenase (GAPDH) (1:2000, Proteintech, China) at 4 °C overnight. Next, Tris-buffered saline containing 0.5% (*w*/*v*) Tween 20 buffer was used to wash membranes three times, and the membranes were incubated with the sheep anti-rabbit or sheep anti-mouse IgG secondary antibodies (1:10,000, LK, China) for 1 h. Subsequently, the protein bands were detected using an efficient chemiluminescence (ECL) kit (Thermo Scientific) following the manufacturer’s protocol. The GAPDH antibody served as a control. The results of the quantitative analysis were assessed using ImageJ software.

### Statistical analysis

Each experiment was performed at least in triplicate. Data were expressed as means ± SEM. The discrepancies in grouping variables were analyzed using the Student *t* test. All statistical analyses were performed using SPSS 20.0, and *P* < 0.05 indicated a statistically significant difference.

## Data Availability

The data sets used and/or analyzed during the current study are available from the corresponding author on reasonable request.

## Abbreviations

ITGBL1: Integrin, beta-like 1
GC: Gastric carcinoma
TNM: Tumor Node Metastasis
EMT: Epithelial–mesenchymal transition
ECM: Extracellular matrix
TIED: Ten β Integrin EGF-like repeat domains
CRC: Colorectal carcinoma
NSCLC: Non-small cell lung cancer
HSPGs: Heparan sulfate proteoglycans
TIMER: Tumor Immune Estimation Resource
TISIDB: Tumor and Immune System Interaction DataBase
qRT–PCR: Quantitative reverse transcription–polymerase chain reaction
TCGA: The Cancer Genome Atlas
OS: Overall survival
TILs: Tumor-infiltrating lymphocytes
GEO: Gene Expression Omnibus
DMEM: Dulbecco’s Modified Eagle medium
7-AAD: 7-Aminoactinomycin D
BCA: Bicinchoninic acid
GAPDH: Glyceraldehyde-3-phosphate dehydrogenase
